# Phytochemical Composition Antioxidant and Anti-Inflammatory Activity of *Artemisia dracunculus* and *Artemisia abrotanum*

**DOI:** 10.3390/antiox13081016

**Published:** 2024-08-20

**Authors:** Mădălina Țicolea, Raluca Maria Pop, Marcel Pârvu, Lia-Oxana Usatiuc, Ana Uifălean, Floricuța Ranga, Alina Elena Pârvu

**Affiliations:** 1 Department of Morpho-Functional Sciences, Discipline of Pathophysiology, Iuliu Haţieganu University of Medicine and Pharmacy, 400012 Cluj-Napoca, Romania; madalina.ticolea@umfcluj.ro (M.Ț.); lia.usatiuc@umfcluj.ro (L.-O.U.); uifaleanana@gmail.com (A.U.); parvualinaelena@umfcluj.ro (A.E.P.); 2 Department of Morpho-Functional Sciences, Discipline of Pharmacology, Toxicology and Clinical Pharmacology, Iuliu Haţieganu University of Medicine and Pharmacy, 400337 Cluj-Napoca, Romania; 3 Department of Biology, Babes-Bolyai University, 400015 Cluj-Napoca, Romania; 4 Food Science and Technology, Department of Food Science, University of Agricultural Science and Veterinary Medicine Cluj-Napoca, Calea Mănăștur, No 3-5, 400372 Cluj-Napoca, Romania; florica.ranga@usamvcluj.ro

**Keywords:** *Artemisia dracunculus*, *Artemisia abrotarum*, inflammation, oxidative stress, antioxidant

## Abstract

This study aimed to investigate the antioxidant and anti-inflammatory activities mechanism of *Artemisia dracunculus* (*A. dracunculus*) and *Artemisia abrotanum* (*A. abrotanum*) ethanol extracts in acute rat inflammation induced in Wistar male rats with turpentine oil. The characterization of the polyphenolic compounds in the extracts was conducted using UV–Vis and Fourier-transform infrared spectroscopy and high-performance liquid chromatography coupled with mass spectrometry techniques. The antioxidant activity of the extracts was evaluated in vitro by DPPH, FRAP, H_2_O_2_, and NO scavenging tests and in vivo by measuring the total oxidative status (TOS), total antioxidant capacity (TAC), oxidative stress index (OSI), 8-hydroxy-deoxyguanosine (8-Oxo-dG), advanced oxidation protein products (AOPP), malondialdehyde (MDA), nitric oxide (NO), 3-nitrotyrosine (3NT), and total thiols (SH). Inflammation was evaluated by measuring nuclear factor-kB-p65 (NfkB-p65) and NLRP3 inflammasome activation with IL-1β, IL-18, and gasdermin D. Liver and renal toxicity was determined following transaminases (ALT and AST), creatinine, and urea. The experimental results indicated that *A. dracunculus* and *A. abrotanum* ethanol extracts have moderate in vitro antioxidant activity and had in vivo antioxidant activity and an anti-inflammatory effect by NfkB-p65, IL-1b, IL-18, and gasdermin D serum level reduction. The antioxidant activity correlated with the chemical composition of the extracts. These results bring evidence-based use of *A. dracunculus* and *A. abrotanum’s* in traditional and contemporary medicine.

## 1. Introduction

In recent years, inflammation has become one of the most important research topics for the study of human diseases. The inflammatory cascade includes a long chain of preprogrammed and stereotyped molecular reactions and cellular responses triggered by cellular and tissue injury to restore tissue integrity [1]. However, if it does not reach the resolution phase, inflammation contributes to further tissue damage, chronic diseases, and possibly organ failure and death [2]. There are large numbers of illnesses related to inflammation, starting with acute and chronic inflammatory diseases and continuing with atherosclerosis, coronary heart disease, diabetes mellitus, obesity, cancer, and many others [3]. During inflammation, macrophages recognize pathogen-associated molecular patterns (PAMPs) or damage-associated molecular patterns (DAMPs) and become activated. One consequence is the activation of the transcription factor NF-κB, which will increase the expression of the pro-inflammatory cytokines, like IL-1β, IL-6, and TNF-α, and inducible nitric oxide synthase (iNOS) with nitric oxide (NO) release [4]. Another consequence is the production of high levels of reactive oxygen species (ROS) and reactive nitrogen species (RNS) as a defense mechanism. An overactive inflammatory response can associate oxidative stress (OS), defined as a disbalance between antioxidants and oxidants, like ROS and RNS, in favor of the latter [5]. Furthermore, oxidative stress can damage biomacromolecules, such as nucleic acids, proteins, lipids, and sugars, leading to oxidative injury and diseases [6,7]. At the same time, ROS may amplify the NF-κB signaling pathway and trigger inflammasome activation [8]. Inflammasomes are a group of cytosolic multiprotein complexes vital in innate immunity because they serve as signaling platforms. Of the several types of inflammasomes that were described, NLRP3 is the best-characterized inflammasome [8]. It is expressed in neutrophils, monocytes, lymphocytes, dendritic cells, osteoblasts, and epithelial cells. In diseases, such as gout, atherosclerosis, type II diabetes, cancers, and others, a pathological NLRP3 inflammasome activation was involved [9,10]. For all these reasons, oxidative stress and the NLRP3 inflammasome have become therapeutic targets in many diseases.

There are a lot of studies showing that antioxidants may be useful to reduce the risk or treat several diseases. Medicinal plants are relevant sources with anti-inflammatory and antioxidant activities for many therapeutic purposes. In the last few years, *Artemisia* species have attracted research attention due to the 2015 Nobel Prize award in medicine for the discovery of artemisinin in *Artemisia annua*, a sesquiterpenoid lactone with an antimalarial effect. The *Artemisia* genus of the *Asteraceae* family includes over 500 species worldwide that are used in traditional and contemporary medicine [11]. Among them, *Artemisia dracunculus* (*A. dracunculus*), *Artemisia abrotanum* (*A. abrotanum*), *Artemisia absinthium, Artemisia annua*, and *Artemisia vulgaris* are the most popular. In folk medicine, these plants are used for antimalarial, antidiabetic, hepatoprotective, neuroprotective, antidepressant, antibacterial, antifungal, cytotoxic, and digestion-stimulating activities [12,13]. Initially, research focused on the plant products’ phytochemicals and later on the pharmacological activities. The phytochemical profiling of genus *Artemisia* plants showed a rich content of monoterpenoids, sesquiterpenoids, coumarins, flavonoids, and sterols [14]. Studies on these species’ chemical composition and bioactivity have confirmed their traditional use and also documented new pharmacological activities [15].

The present study aimed to provide insights into the *A. dracunculus* and *A. abrotanum* ethanol extracts phytochemical analysis, antioxidant, and anti-inflammatory activity in a rat turpentine-oil-induced inflammation. The phytochemical analysis was performed by measuring TPC, TFC, and polyphenols by HPLC-DAD-ESI MS and FTIR. The antioxidant activity was evaluated in vitro by DPPH, FTIR, H_2_O_2_, and NO scavenging activity. In vivo, antioxidant activity was appreciated by measuring serum oxidative stress biomarkers, including TOS, TAC, OSI, 8-OHdG, AOPP, MDA, NO, 3NT, and SH. To evaluate the anti-inflammatory effect, the level of NfkB-p65 was also analyzed as was the NLRP3 inflammasome activation by measuring IL-1b, IL-18, and gasdermin D. To our best knowledge, these mechanisms have not previously been documented.

## 2. Materials and Methods

### 2.1. Chemicals

Methanol (CH_3_OH), ethanol (C_2_H_5_OH), acetonitrile (CH_3_CN), diethylether (C_2_H_5_OC_2_H_5_), vanadium (III) chloride (VCl_3_), hydrochloric acid (HCl), ammonium iron (II) sulfate ((NH_4_)_2_Fe(SO_4_)_2_·6H_2_O), sulfanilamide (C_6_H_8_N_2_O_2_S), N-(1-naphthyl)ethylenediamine dihydrochloride (C_12_H_14_N_2_·2HCl), xylenol orange [o-cresosulfonphthalein-3,3-bis(sodium methyliminodiacetate)], sulfuric acid (H_2_SO_4_—with sulfur in the +6 oxidation state), acetic acid (CH_3_COOH), glacial acetic acid (CH_3_COOH), glycerol (C_3_H_8_O_3_), ortho-dianisidine dihydrochloride (3-3′-dimethoxybenzidine), hydrogen peroxide (H_2_O_2_), thiobarbituric acid (C_4_H_4_N_2_O_2_S), o-phthalaldehyde (C₆H_4_(CHO)_2_), aluminum chloride (AlCl_3_), 1-ethyl-3-methylimidazolium chloride (C₆H_11_ClN_2_), sodium nitrite (NaNO_2_), sodium carbonate (Na_2_CO_3_), sodium hydroxide (NaOH), sodium nitroprusside (Na_2_[Fe(CN)_5_NO]), chloramine-T (C_7_H_7_ClNNaO_2_S), potassium iodide (KI), trichloroacetic acid (CCl_3_COOH), Folin-Ciocalteu′s phenol reagent (C_10_H_7_NaO_3_S·5H_2_O, and 5,5′-dithio-bis-(2-nitrobenzoic acid) (C_14_H_8_N_2_O_8_S_2_) were purchased from Merck (Darmstadt, Germany); chlorogenic acid (C_16_H_18_O_9_), luteolin (C_15_H_10_O_6_), rutin (C_27_H_30_O_16_), gallic acid (C_7_H_6_O_5_), and quercetin (C_15_H_10_O_7_) analytical standards were obtained from Sigma (St. Louis, MO, USA); trolox (6-hydroxy-2,5,7,8-tetramethylchroman-2-carboxylic acid) (C_14_H_18_O_4_) was purchased from Alfa-Aesar (Karlsruhe, Germany); rat ELISA kits were purchased from Elabscience Bionovation Inc. (Houston, TX, USA) and MyBiosource (San Diego, CA, USA); and transaminases ALT and AST, urea, and creatinine reagents were purchased from BioSystems Diagnostic (Ilfov, Romania). All chemicals were ultrapure grade. Ultrapure water for the HPLC analysis was purified using the Direct-Q UV system from Millipore (Burlington, MA, USA).

### 2.2. Plant Material Collection and Extract Preparation

In June 2021, *A. dracunculus* (Voucher 670072) and *A. abrotanum* (Voucher 670073) aerial parts were obtained from the Botanical Garden “Alexandru Borza” in Cluj-Napoca (46°45′36″ N and 23°35′13″ E). P.M. from Babes-Bolyai University of Cluj-Napoca taxonomically identified the plant materials. By using the modified Squibb cold repercolation method, fresh *A. dracunculus* and *A. abrotanum* aerial parts (fragments of 0.5–1 cm) (400 g) were extracted with 70% ethanol (1.2 L) over 3 days at room temperature conditions. The final extracts were *A. dracunculus* 1:1.3 g/mL (*w*:*v*) and *A. abrotanum* 1:1.1 g/mL (*w*:*v*) [16].

### 2.3. Phytochemical Analysis

#### 2.3.1. Total Polyphenol Content

*A. dracunculus* and *A. abrotanum* extracts’ total polyphenol content (TPC) was determined using a modified Folin–Ciocâlteu method. Briefly, 2 mL of *A. dracunculus* or *A. abrotanum* extracts were diluted 25-fold, followed by 1 mL of Folin–Ciocâlteu reagent and 10.0 mL of distilled water addition. The mixture was then brought to a final volume of 25 mL using a (290 g/L) sodium carbonate solution. The absorbance of the samples was measured at 760 nm after 30-min incubation in the dark. The TPC values were then reported as milligrams of gallic acid equivalents (GAE) per gram of dry-weight (d.w.) plant material (mg GAE/g d.w. plant material) [16].

#### 2.3.2. Total Flavonoid Content

*A. dracunculus* and *A. abrotanum* extracts’ total flavonoid content (TFC) was determined following the previously described method [17]. Briefly, 1 mL from both extracts was initially mixed with 5% NaNO_2_ (0.3 mL), 10% AlCl_3_ (0.3 mL), and 1 M NaOH (2 mL) solution. Finally, distilled water was added to the mixture until it reached a final volume of 10 mL. The absorption was recorded at 510 nm after a 15-min incubation. TFC was expressed as mg quercetin equivalents (QE) per 100 g of dry-weight plant material (mg QE/100g d.w. plant material).

#### 2.3.3. High-Performance Liquid Chromatography Coupled with Electrospray Ionization Mass Spectrometry (HPLC-ESI MS) Analysis

High-performance liquid chromatography (Agilent 1200 HPLC) with DAD detection, coupled to a single quadrupole mass spectrometer (Agilent 6110 MS, SpectraLab Scientific Inc., Markham, ON, Canada), was used to characterize the *A. dracunculus* and *A. abrotanum* ethanol extracts. Separation of the compounds was carried out at room temperature using an XDB C18 Eclipse column (4.6 × 150 mm, particle size 5 μm) (Agilent Technologies, Santa Clara, CA, USA).

The phenolic compounds of the *A. dracunculus* and *A. abrotanum* ethanol extracts were determined as previously described with some modifications. A gradient program was employed with mobile phase A consisting of 0.1% acetic acid/acetonitrile (99:1) in distilled water (*v*/*v*) and mobile phase B consisting of 0.1% acetic acid in acetonitrile (*v*/*v*). Starting at 95% A (0–2 min), the elution program decreased to 95–60% A (2–18 min), 60–10% A (18–20 min), and 10% A (20–24 min). In the end, mobile phase A was raised back to 95% in less than one minute and kept there for five more minutes [18]. A 0.5 mL/min flow rate was employed. The absorbance spectrum was recorded at 280 nm (specific for phenolic acids) and at 340 nm (specific for flavonoids). After the compounds were separated, they were directed into the MS, where the ESI source was used in the (+) mode to ionize the molecules. The nitrogen flow was set to 8 L/min, the temperature was set at 350 °C, and the capillary voltage was set at 3000 V. The compounds were scanned at intervals of 100–1000 *m*/*z*.

Data analysis was conducted using Agilent ChemStation Software (Rev B.04.02 SP1, Palo Alto, CA, USA). The identification of phenolic compounds was performed using retention time, UV−visible spectra, mass spectra, co-chromatography with standards (when available), the literature data, and the Phenol-Explorer database. The lyophilized extract was dissolved in MeOH before LC analysis [18].

Five different concentrations of standard solutions dissolved in methanol were injected to construct calibration curves for quantification.

For phenolic acids, quantification was performed using chlorogenic acid (R^2^ = 0.9937, LOD = 0.41 μg/mL, LOQ = 1.64 μg/mL); for flavones, luteolin (R^2^ = 0.9972, LOD = 0.26 μg/mL, LOQ = 0.95 μg/mL); and for flavonols, rutin (R^2^ = 0.9981, LOD = 0.21 μg/mL, LOQ = 0.84 μg/mL).

#### 2.3.4. Fourier-Transform Infrared Spectroscopy (FTIR) Analysis

The FTIR analysis of *A. dracunculus* and *A. abrotanum* ethanol extracts was performed using a (SHIMADZU CORPORATION, Tokyo, Japan) equipped with attenuated total reflectance (ATR) and a Zinc Selenide (ZnSe) Composite as an internal reflection accessory. The extracts were pipetted directly onto the crystal and allowed to evaporate. The spectra were then registered between 4000–650 cm^−1^, with an air spectrum used as the background [17].

### 2.4. In Vitro Antioxidant Activity Analysis

#### 2.4.1. 2,2-Diphenyl-1-Picrylhydrazyl (DPPH) Radical Scavenging Capacity

The DPPH radical scavenging capacity of *A. dracunculus* and *A. abrotanum* extracts was measured according to a method previously described [17]. Briefly, 3 mL of the extract was mixed with 1 mL of DPPH and a 0.1 mM methanol solution. Absorbance was measured after 30 min of incubation (room temperature, dark conditions) at 517 nm. Using the formula AA% = [(A control − A sample)/A control] × 100, the percentage of radical scavenging activity (AA%) was calculated and then converted to μg Trolox equivalent per gram of dry weight plant material (μgTE/g d.w. plant material). Finally, DPPH radical scavenging activity was expressed as IC50 (half maximal inhibitory concentration) in μgTE/mL. An IC50 ≤ 50 μg/mL indicates good antioxidant capacity, an IC50 within the range of 50–100 μgTE/mL indicates moderate antioxidant capacity, and an IC50 ≥ 200 μgTE/mL indicates negligible antioxidant capacity [19].

#### 2.4.2. Ferric Reducing Antioxidant Power (FRAP) Assay

The FRAP assay was used to calculate the reduction capacity of the *A. dracunculus* and *A. abrotanum* extracts [17]. Briefly, 100 μL of each ethanol extract sample was mixed thoroughly with 3.4 μL of FRAP reagent. Absorbance was measured at 593 nm after 30 min, and the results were expressed as IC50 in μg Trolox equivalent mL (μgTE/mL).

#### 2.4.3. Hydrogen Peroxide (H_2_O_2_) Scavenging Activity

The determination of the *A. dracunculus* and *A. abrotanum* ethanol extracts’ ability to scavenge hydrogen peroxide (H_2_O_2_) was performed as previously described [19]. Briefly, the extracts were mixed with an H_2_O_2_ solution, and absorbance was measured at 230 nm against a phosphate buffer blank after 10 min. Using the formula % scavenged H_2_O_2_ = (A control − A sample/A control) × 100, the percentage of H_2_O_2_ scavenging was calculated. The results were then expressed as IC50 in μg Trolox equivalent per mL (μgTE/mL).

#### 2.4.4. The Nitric Oxide (NO) Radical Scavenging Assay

As previously described, for the nitric oxide radical scavenging assay [19] sodium nitroprusside was used to generate nitric oxide (NO), which was then measured by using the Griess reagent. In summary, 0.5 mL of *A. dracunculus* and *A. abrotanum* extracts were mixed with a sodium nitroprusside solution (2 mL SNP and 0.5 mL PBS, pH 7.4) and incubated for 2.5 h at 25 °C. Next, to 0.5 mL of this was mixted with 1 mL of sulphanilic acid, and after 5 min, 1 mL of Naphthylethylene-diamine-dihydrochloride was added. The resulting mixture was vortexed and incubated in the dark for 30 min. The absorbance was measured at 546 nm, and the percentage of inhibition was determined using the formula: % scavenged NO = (A blank − A sample/A blank) × 100. The results were presented as IC50 in μg quercetin equivalent per mL (μg QE/ mL).

All in vitro antioxidant assays were conducted in triplicate. The measurements were carried out using a UV–Vis spectrophotometer (Jasco V-350, Jasco International Co., Ltd., Tokyo, Japan).

### 2.5. In Vivo Experimental Design

#### 2.5.1. Animal Subjects

Adult albino Wistar male rats weighing 200–250 g were obtained from the Establishment for Breeding and Use of Laboratory Animals of the “Iuliu Hațieganu” University of Medicine and Pharmacy, Cluj-Napoca, Romania. Rats were accommodated in standard polypropylene cages, in standard laboratory conditions (temperature 25 ± 1 °C. relative humidity 55 ± 5%. and 12 h light/dark cycle). They had free access to a standard granular diet and ad libitum water. All the procedures comply with Directive 2010/63/EU and Romanian national law 43/2014 for the protection of animals used for scientific purposes. The project was approved by the Veterinary Sanitary Direction and Food Safety Cluj-Napoca (No. 303/04.04.2022). The experiments were performed in triplicate.

#### 2.5.2. Experimental Protocol

The animals were randomly distributed into 10 groups (n = 9). On the first day, inflammation was induced with turpentine oil (6 mL/kg b.w. by intramuscular injection (i.m.)), excepting the negative control group (CONTROL). Then, starting from the first day, for 10 days animals were treated orally by gavage as follows: the CONTROL and inflammation group (INFL) were treated with tap water (1 mL/rat/day(d)); the anti-inflammatory drug group was treated with diclofenac (10 mg/kg b.w./d) (DICLO); *A. dracunculus* experimental groups were treated with three dilutions of the plant extract (100%. 50% and 25%) (AD100%, AD50%, AD25%) (1 mL/rat/d); *A. abrotanum* experimental groups were treated with three dilutions of the plant extract (100%. 50% and 25%) (AA100%, AA50%, AA25%) (1 mL/rat/d); and the antioxidant control group was treated with Trolox (50 mg/kg b.w./d) (TX). On the 11th day, animals were anesthetized by using ketamine (60 mg/kg b.w.) and xylazine (15 mg/kg b.w.) [20], blood was withdrawn by retro-orbital puncture, and serum was separated and stored at −80 °C until use.

#### 2.5.3. Oxidative Stress Marker Assessment

##### Total Oxidative Status (TOS)

The total oxidative status (TOS) was used to measure the oxidation of ferrous ions to ferric ions in the presence of ROS in an acidic medium. The results were expressed in μmol H_2_O_2_ equivalent/L (μmol H_2_O_2_E/L) [21].

##### Total Antioxidant Capacity (TAC)

The total antioxidant capacity (TAC) was used to measure the rate of hydroxyl radical production suppression by the Fenton reaction by the anti-oxidants present in the serum [22]. The results are expressed as mmol Trolox equivalent/L (mmol TE/L).

##### Oxidative Stress Index (OSI)

The oxidative stress index (OSI) was used to evaluate the ratio the TOS to the TAC [23]: OSI (Arbitrary Unit) = TOS (mol H_2_O_2_ Equiv/L)/TAC (mmol Trolox Equiv/L). It is an indicator of the degree of oxidative stress.

##### 8-Hydroxydeoxyguanosine (8-OHdG)

8-hydroxydeoxyguanosine (8-OHdG), a biomarker of oxidative DNA damage, was detected by using an ELISA kit (E-EL-0028) according to the manufacturer’s instructions [24]; results are expressed as ng/mL.

##### Advanced Oxidation Protein Products (AOPP)

The advanced oxidation protein products (AOPP), a biomarker of protein oxidation, were measured by a spectrophotometric method [25]. Briefly, samples and chloramine T as blank were diluted to 10% in PBS. Afterward, potassium iodide and glacial acetic acid were added. The absorbances of the samples were recorded at 340 nm after glacial acetic acid was added. The subtraction of the optical density of the blank sample was performed before data analysis. AOPP concentrations were expressed in µmol chloramine-T equivalent/L (µmol chloramineE/L).

##### The Malondialdehyde (MDA)

The malondialdehyde (MDA), a lipid peroxidation marker, was measured by using thiobarbituric acid as previously described. Briefly, 0.1 mL of serum was mixed with 0.1 mL of 40% trichloroacetic acid, which was further mixed with 0.2 mL of 0.67% thiobarbituric acid. The mix was placed in a boiling water bath for 30 min, after which it was cooled in an ice water bath. After cooling, the mix was centrifuged for 5 min at 3.461 g. The supernatant absorbance was recorded at 532 nm and the serum MDA concentration was expressed as nmol/mL of serum [26].

##### Nitric Oxide Synthesis (NO)

Nitric oxide synthesis (NO) was indirectly determined by measuring total nitrites and nitrates by using the Griess reaction. Briefly, serum proteins were removed by extraction with a 3:1 (*v*/*v*) solution of methanol/diethyl ether, nitrates were reduced to nitrites by adding vanadium (III) chloride, and then the Griess reagent was added. Sample absorbance was read at 540 nm and the results were expressed as nitrite μmol/L [27,28].

##### 3-Nitrotyrosine (3NT)

The 3-nitrotyrosine (3NT), a relatively specific marker of oxidative damage mediated by peroxynitrite [29], was measured by using an ELISA kit (E-EL-0040, Elabscience Innovation Bionovation Inc., Houston, Texas, USA) according to the manufacturer’s instructions; the results were expressed as ng/mL.

##### Total Thiols (SH)

The total thiols (SH), an antioxidant marker, was measured by using Ellman’s reagent (Sigma-Aldrich, Munich, Germany) [30]. Supernatant absorbance was read at 412 nm and serum SH concentration was expressed as mmol glutathione/mL (mmol GSH/mL).

The spectroscopic analysis (TOS, TAC, OSI, AOPP, MDA, NO, and SH) was performed using a UV–Vis spectrophotometer (Jasco V-350, Jasco International Co., Ltd., Tokyo, Japan). The ELISA analysis was performed using a Biotek Microplate 50 TS washer (Agilent Technologies Inc., Santa Clara, CA, USA) and an 800 TS ELISA microplate reader (Agilent Technologies Inc., Santa Clara, CA, USA).

#### 2.5.4. Inflammatory Marker Assessment

The anti-inflammatory activity was assessed by measuring serum nuclear factor Kappa B p65 (NfkB-p65) (E-EL-RO674), interleukin 1 beta (IL-1β) (E-EL-0012), interleukin 18 (IL-18) (E-EL-R0567), and gasdermin D (GSDMD) (MBS2705517) with ELISA kits according to the manufacturer’s instructions. For NfkB-p65 and GSDMD, the results were expressed as ng/mL, and for IL-1β and IL-18, the results were expressed as pg/mL. The equipment used for ELISA analysis was previously described.

#### 2.5.5. Toxicity Assessment

Liver toxicity was also evaluated by measuring alanine transaminase (ALT) and aspartate aminotransferase (AST) and renal toxicity was measured by measuring urea and creatinine. The Jasco V-530 UV–Vis spectrophotometer (Jasco International Co. Ltd., Tokyo, Japan) was used for their determination following the commercially available kit instructions.

### 2.6. Statistical Analysis

The results were displayed as mean ± standard deviation (SD) whenever data were normally distributed. The groups that underwent the experiment were compared by using the one-way analysis of variance (ANOVA) and the post hoc Bonferroni–Holm tests. The Pearson test and principal component analysis (PCA) were used for correlation analysis. A *p* < 0.05 was considered to be statistically significant. Statistical analyses were performed using SPSS Statistics Version 26.0 for Windows (SPSS, Chicago, IL, USA) and GraphPad Prism Version 8.0 (GraphPad Software, San Diego, CA, USA).

## 3. Results

### 3.1. Phytochemical Analysis

#### 3.1.1. Total Polyphenol and Flavonoid Content

TPC and TFC of the *A. dracunculus* and *A. abrotanum* ethanol extracts were significant, with *A. dracunculus* having a higher TPC and *A. abrotanum* having a higher TFC (Table 1).

#### 3.1.2. High-Performance Liquid Chromatography Coupled with Electrospray Ionization Mass Spectrometry (HPLC-ESI MS) Analysis

**Figure 1:**
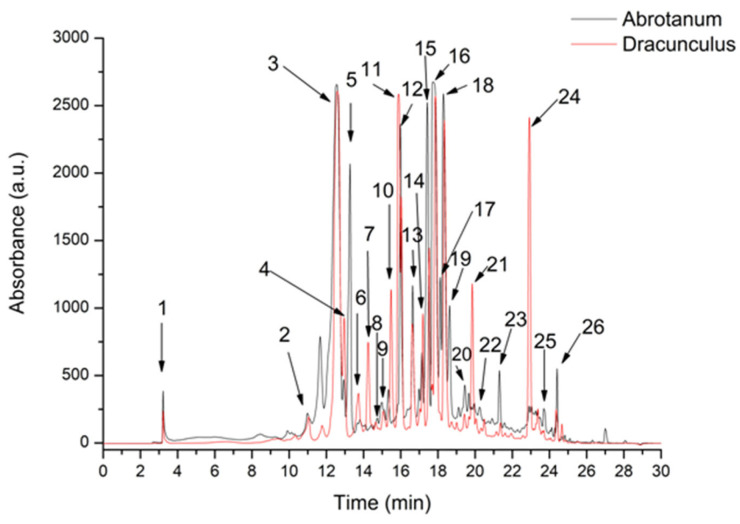
HPLC chromatogram of phenolic compounds from *A. abrotanum* and *A. dracunculus* ethanolic extracts. The peak identification is provided in Table 2.

Phenolics HPLC-ESI MS analysis showed that ethanol extracts were rich in non-flavonoid compounds, like hydroxycinnamic and hydroxybenzoic acids (Figure 1; Table 2). Among them, 5-Feruloylquinic acid was found exclusively in the *A. dracunculus* (1/4 of the total phenolic compounds) and two phenolic acids, Caffeoyl acid-glucoside and Quinic acid, were found exclusively in *A. abrotanum*, providing a different pattern of response.

#### 3.1.3. Fourier-Transform Infrared Spectroscopy (FTIR) Analysis

**Figure 2:**
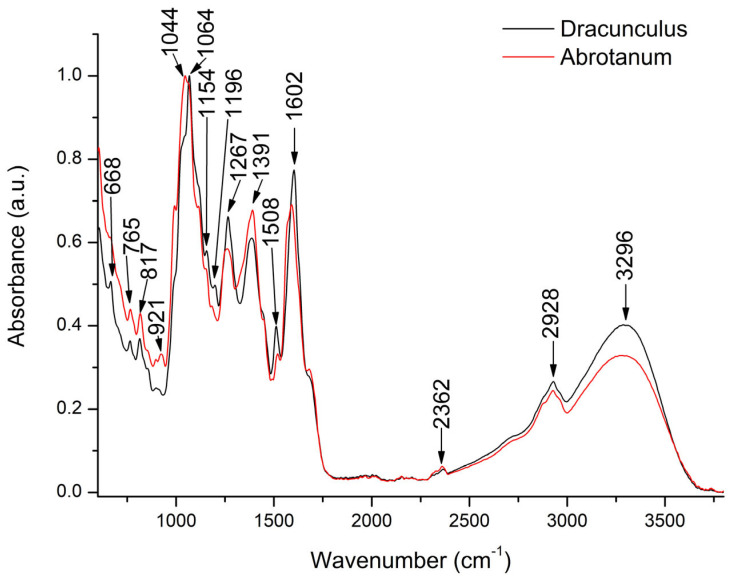
Comparative general FTIR spectra of *A. dracunculus* and *A. abrotanum* ethanol extracts (600–3500 cm^−1^).

Using FTIR analysis, the general composition of the compounds can be assessed based on the signals given by the chemical bonds and functional groups existing in the molecules of plant extract [17,31]. Figure 2 presents the comparative FTIR screening of bioactive compounds present in *A. dracunculus* and *A. abrotanum* ethanol extracts.

A similar FTIR profile could be observed for both plant extracts, with some differences in peak intensities (Figure 2). Thus, in the region between 3000–4500 cm^−1^, characterized by the intense absorption peak at 3296 cm^−1^, *A. dracunculus* extract showed higher absorption intensity. This can indicate a higher concentration of compounds containing the OH group. The deformation vibrations of OH can be assigned to molecules with aromatic carbons, alcoholic functions, phenolic -OH stretching existing in the phenolic compounds, or different glycoside derivatives [32,33]. Next, *A. dracunculus* showed a slightly high-intensity peak at 2928 cm^−1^, which can be attributed to the C-H vibrations in the alkyl groups or to the -CH_2_ and -CH_3_ vibrations in the carbohydrates or polyphenols [32]. The fingerprint region ranging from 1800–600 cm^−1^ is characterized by several intense peaks, with slight intensity variations between the two *Artemisia* extracts. Thus, the band at 1602 cm^−1^ is highly represented in both samples and can be assigned either to C=O or conjugated C=C stretching vibration specific for phenolic acids derivatives or aromatic ring of phenolic compounds in the extract. Also, this absorption band can be assigned to the vibrations of C=O and C=C bonds existing in the phenolic compounds. Next, the C-C stretching of the aromatic rings is evidentiated by the absorption band at 1508 cm^−1^ [32]. The peak at 1392 cm^−1^ can be attributed to the C-H stretching vibration [33] or aromatic compounds while the intense peak at 1044 cm^−1^ could be assigned to the C-O-C stretching vibration in polysaccharides [32]. Furthermore, the high-intensity peak at 1064 cm^−1^ can be assigned to flavonoids or carbohydrates in the *Artemisia* extracts. Finally, the peaks between 900–750 cm^−1^ can be assigned to the C-H out-of-plane bending vibrations in aromatic compounds [34,35,36].

### 3.2. In Vitro Antioxidant Activity

The ethanolic extracts of the *A. dracunculus* and *A. abrotanum* displayed in vitro antioxidant activity. DPPH radical scavenging capacity, FRAP, and H_2_O_2_ scavenging capacities were moderate and smaller than those of TE, and NO scavenging activity was also moderate and smaller than that of quercitin (Table 3).

To evaluate OS, global and specific biomarkers were used. In INFL animals there was a high OS as compared to the CONTROL because TOS and OSI were significantly increased (*p* < 0.001) and TAC was significantly reduced (*p* < 0.001). By analyzing the specific OS biomarkers, we found that 8-OHdG, NO, and 3NT were more elevated (*p* < 0.001) than AOPP (*p* < 0.01) and MDA (*p* < 0.05). SH decreased (*p* < 0.01) as it is involved in endogenous antioxidant reduction (Table 4).

Diclofenac treatment reduced TOS and OSI (*p* < 0.01). This effect was associated with a significant decrease in NO, 3NT, and MDA (*p* < 0.001) and 8-OhdG and AOPP (*p* < 0.01). Trolox antioxidant activity caused TOS, OSI, 8-OHdG, 3NT, and AOPP reduction (*p* < 0.001) and the effect was better than that of diclofenac (Table 4).

Treatment with AD 100% and AD 50% lowered TOS and OSI (*p* < 0.001) by causing an important reduction in 8-OhdG, NO, and 3NT (*p* < 0.001) and a moderate decrease in AOPP (*p* < 0.01) and MDA (*p* < 0.01). AD 25% caused the reduction in 8-OHdG, 3NT (*p* < 0.001), and MDA (*p* < 0.05). TAC and SH were not influenced by AD 100%, AD 50%, or AD 25%. Diclofenac’s antioxidant effect was better than that of AD only on 8-OHdG and MDA. AD dilutions were less effective than TX on TOS, OSI, MDA, and NO (Table 4).

The AA extract dilutions lowered significantly with regard to TOS and OSI (*p* < 0.01) but the effect was smaller than that of AD extract dilutions. For the AA extract, 8-OHdG and 3NT (*p* < 0.001) were significantly decreased. On NO, AOPP, and MDA, AA had a lower antioxidant effect (*p* < 0.01). TAC was not influenced by all AA dilutions but SH was increased only by AA 100% (*p* < 0.01).

Diclofenac and TX had a better inhibitory effect than AA dilutions only on TOS and OSI (*p* < 0.01) and AA reduced more 8-OhdG than diclofenac (Table 4).

### 3.3. In Vivo Anti-Inflammatory Activity

For the anti-inflammatory activity assessment, NF-kB-p65, IL-1b, IL-18, and gasdermine D were measured to evaluate inflammasome NLRP3 activation. In the INFL group, NF-kB-p65, IL-1b, IL-18, and gasdermine D were significantly increased (*p* < 0.001). Both extracts of AD and AA lowered NF-kB-p65 significantly (*p* < 0.001) and this effect was smaller than that of diclofenac and TX (*p* < 0.05) (Table 5).

IL-1b was reduced very significantly by AD 100% and AD 50%AA and this effect was comparable to those of diclofenac and TX. AA 25% had a lower inhibitory effect on IL-1b (*p* < 0.05). All AA dilutions caused a significant reduction in IL-1b (*p* < 0.001) and there were no significant differences between AA, diclofenac, and TX activity on IL-1b (*p* > 0.05) (Table 5).

AD had a dose-dependent effect on IL-18, AD 100% and AD 50% very much reduced IL-18 (*p* < 0.001), and AD 25% caused a smaller decrease (*p* < 0.05). The effects of AD 100% and AD 50% on IL-18 were comparable to those of diclofenac and TX. AA caused a very significant reduction in IL-18 for all dilutions (*p* < 0.001) and the effects were like those of diclofenac and TX (Table 5).

AD and AA dilutions had a small inhibitory activity on GSDMD (*p* < 0.05), and these effects were smaller than those of diclofenac and TX (Table 5).

### 3.4. PCA Analysis

**Figure 3:**
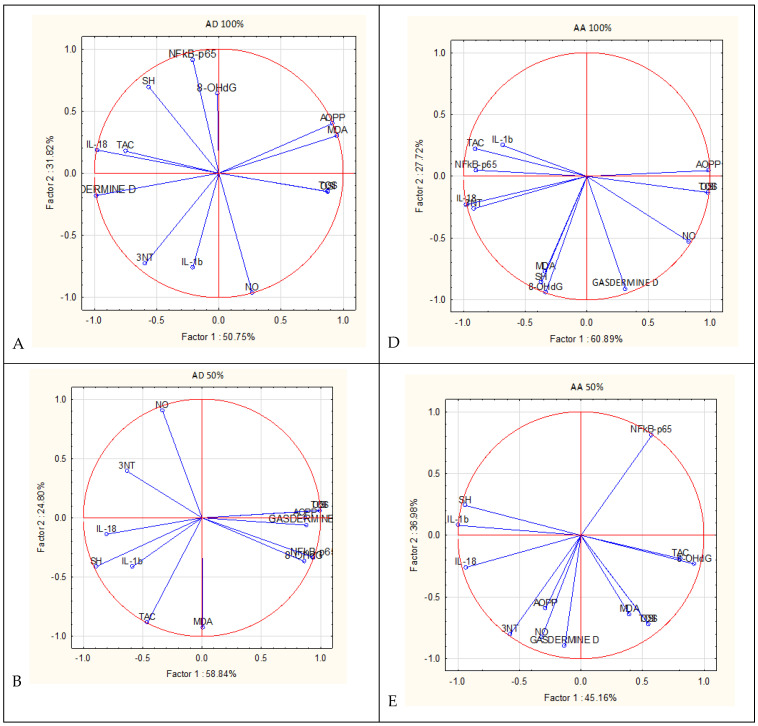

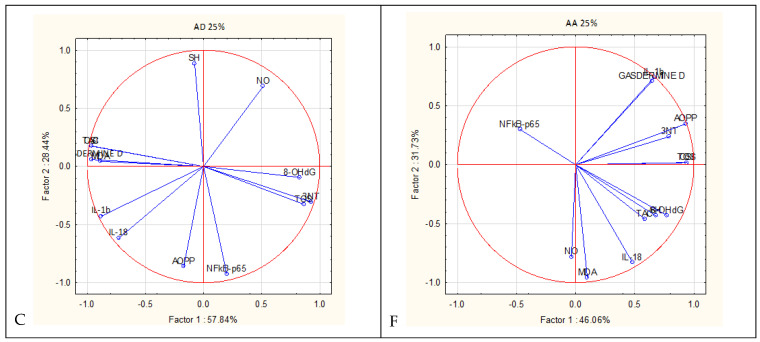
The PCA results of oxidative stress and inflammatory biomarkers based on the correlation matrix with PC1 and PC2 for *A. dracunculus* and *A. abrotanum* ethanol extracts: (**A**) PCA of AD 100%; (**B**) PCA of AD 50%; (**C**) PCA of AD 25%; (**D**) PCA of AA 100%; (**E**) PCA of AA 50%; (**F**) PCA of AA 25%. AD 100%—*A. dracunculus* 100%; AD 50%—*A. dracunculus* 50%; AD 25%—*A. dracunculus* 25%; AA 100%—*A. abrotanum* 100%; AA 50%—*A. abrotanum* 50%; AA 25%—*A. abrotanum* 25%.

The PCA analysis was performed to establish the correlation between analyzed parameters and to check their variability in the rat groups according to different concentrations in plant extract administration (Figure 3). The variability of these parameters was explained by the comparisons of the first principal component (PC1) and the second one (PC2), as shown in the score plots (Figure 3). These components (PC1 and PC2) explained 82.57%, 83.64%, and 86.28% of the total variance (Figure 3A–C, respectively) in the case of *A. dracunculus* administration (100%, 50%, and 25% plant extract). In the case of *A. abrotanum* administration (100%, 50%, and 25% plant extract), the first two components (PC1 and PC2) explained 88.61%, 82.14%, and 77.79%, respectively (Figure 3D, Figure 3E, and Figure 3F, respectively). It can be observed that the correlations between inflammatory and oxidative stress parameters vary according to the administrated plant extract concentrations. As stated before, the inflammatory markers were significantly increased in the inflammation group and decreased in the treatment groups (as seen in Table 4 and Table 5). The PCA analysis indicated a poor correlation between gasdermin D and NF-kB-p65 in almost all experimental variants (Figure 3A,C–F), except for AD 50% (Figure 3B), suggestive of the extract’s anti-inflammatory effects. The different degrees of correlation between parameters (given by the small angle of the two vectors) suggest the different mechanisms of actions of the plants in accordance with their concentrations. The negative correlation between parameters is given when the vectors diverge and form a large angle, close to 180°. For instance, NO is negatively correlated with IL1b and IL-18 for AD25 and with IL1b for AA25 and AA100, suggesting higher NO values in these groups. These negative correlations can offer insights regarding the capacity of these plant extracts in helping to restore the equilibrium between RNS formation and inflammation.

### 3.5. Toxicity Assessment

The liver injury biomarkers AST and ALT were not significantly increased by inflammation induction and treatments with AD, AA diclofenac, and TX did not influence transaminase concentration (*p* > 0.05) (Table 6).

Inflammation caused a small rise in creatinine and urea (*p* < 0.05). The results of the present study showed the absence of toxic effects of AD and AA on rat kidneys because there was no significant effect on kidney biomarkers, namely creatinine and urea. Diclofenac and TX have no effect either (Table 6).

## 4. Discussion

In this study, we provided new insights regarding the antioxidative and anti-inflammatory effects of the ethanol extracts of *A. dracunculus* and *A. abrotanum*, in correlation with the phytochemical characteristics.

The antioxidant activities of plants are mainly due to the presence of polyphenols, secondary metabolism products of plants that can protect from aging and oxidative stress-induced diseases, like cancer, cardiovascular diseases, diabetes mellitus, and others. Therefore, it is intense work to identify and characterize effective natural plant-derived antioxidants [37].

The combination of ethanol and water is a good extracting solvent for phenolic compounds [38]. In this paper, we demonstrated that *A. dracunculus* ethanolic extract TPC (2.26 ± 0.21 mg GAE/g dw plant material) was higher than in *A. dracunculus* aqueous extract TPC (0.146 ± 0.012 mg GAE/g dw plant material) [39] but smaller than in another *A. dracunculus* ethanol extract (144.28 ± 1.87 mg GAE/g dw plant material) [40]. In the Baiceanu et al. study, *A. abrotanum* TPC was also higher (12.7 ± 0.44 mg GAE/g dw plant material) than in our *extract* (2.10 ± 0.09 mg GAE/g dw plant material) [41]. Furthermore, in this study, *A. dracunculus* TPC was higher than that of *A. abrotanum*.

Flavonoids, non-volatile compounds with important pharmacological effects, were evaluated by measuring the TFC. *A. dracunculus* ethanolic extract TFC (181.50 ± 32.10 mg QE/100 g d.w. plant material) was higher than in other studies (48.84 ± 2.04 mg RE/g DW) [42]. The same was found for *A. abrotanum*, which had a higher TFC (233.15 ± 18.64 mg QE/100 g d.w. plant material) than that found by Baiceanu et al. (6.74 ± 0.32 RE/100 g DW) [41]. In this study, *A. abrotanum* TFC was higher than that of *A. dracunculus*.

These differences between the TPC and TFC of *Artemisia* extracts were considered to be due to the genetic, environmental, climate, and preparation conditions [43]. Moreover, a study analyzing *Artemisia* plants from Central Lithuania showed that TPC and TFC vary depending on different vegetation stages [43].

Due to the complex nature of the phytochemicals, spectrophotometric methods are not sensitive enough to assess bioactive components [44]; HPLC-DAD-ESI MS and FTIR methods were further used for the phytochemical characterization of the plant extracts [45].

Upon HPLC-DAD-ESI MS analysis, 26 phenolic compounds were detected in *A. dracunculus* and A. abrotanum ethanol extracts. The identified phenolic compounds belong to the phenolic acids group (caffeoylquinic and hydroxycinnamic acids) and flavonoids (flavonols and flavones), respectively. Like in other studies [43], the compounds with the highest concentration in both extracts were chlorogenic acid, 3,5-dicaffeoylquinic acid, 4,5-dicaffeoylquinic acid, 3,4-dicaffeoylquinic acid, and rutin. There were *high concentrations of 5-Fe*ruloylquinic acid and 3,4,5-Tricaffeoylquinic acid in *A. dracunculus extract* and of Caffeoyl acid-glucoside in *A. abrotanum* extract. FT-IR analysis confirmed the presence of the polyphenols as identified by HPLC analysis.

Antioxidant activity is the property of antioxidants to inhibit oxidation reactions. The phytochemical composition suggested good antioxidant activity for the *A. dracunculus* and A. abrotanum ethanol extracts because the antioxidant activity depends on the TPC and TFC [38]. Polyphenols act as antioxidants via the scavenging of free radicals and the chelation of transition metals, mediation, and inhibition of enzymes [3]. The in vitro antioxidant activity of *A. dracunculus* and *A. abrotanum* ethanol extracts was evaluated by using four tests, namely DPPH, FRAP, H_2_O_2_, and NO scavenging capacities (Table 3). Both extracts showed moderate antioxidant activity. Other studies testing *A. dracunculus* aqueous extract found a better DPPH free radical inhibitory capacity (IC_50_ 10.71 ± 0.01 μg/mL) compared with Trolox (IC_50_ 5.7 ± 0.92 μg/mL) [39] and for *A. Abrotanum* ethanol *extract*, DPPH (IC_50_ 284.5 ± 16.21 μg/mL) compared with ascorbic acid (IC_50_ 17.34 ± 0.43 μg/mL) was higher [41]. Moreover, *A. abrotanum* DPPH and FRAP showed variability during different vegetation stages [43].

Even if the in vitro antioxidant activity showed good results, further in vivo pharmacological tests were conducted to evaluate the antioxidant and anti-inflammatory effects and the correlation between them.

In folk medicine, *A. dracunculus* is widely used as a spice and it has a long history as a choleretic, antitumoral, and antibacterial agent [45]. Today, due to its antioxidant capacity, high availability, low cost, and low rate of side effects, *A. dracunculus* is considered a potential anti-inflammatory drug [46]. In traditional European medicine, *A. abrotanum* has been successfully used most of all in liver and biliary tract diseases, cancer, children oxyurosis, and ascariasis, as an antipyretic, as a hair growth agent [47], and in the treatment of malaria [48]. Related to *A. abrotanum*, the European Medicines Agency published many books about homeopathic preparations and French Pharmacopoeia included *A. abrotanum* in homeopathic medicine [15,43].

According to the World Health Organization (WHO), about 80% of the world’s population utilizes traditional medicine with herbal extracts for their primary health care. Many of these plant products are natural antioxidant sources that can be used in preventive medicine [2].

Normal aerobic cellular metabolism produces ROS, like superoxide anion, hydrogen peroxide, hydroxyl radical, and organic peroxides, that play crucial roles in cell signaling pathways. During inflammation, leukocytes and mast cells from the injured tissue generate higher quantities of ROS and also NO, which can further produce other reactive nitrogen species (RNS). Moreover, ROS can produce other additional reactive species. In normal conditions, there is an equilibrium between ROS and RNS formation and endogenous enzymatic and nonenzymatic antioxidants. OS occurs due to either an increase in ROS/RNS generation and/or a decrease in the antioxidant defense system. Therefore, the evaluation of OS has to include the determination of secondary products of ROS/RNS and also antioxidant status. TOS represents the measure of the total pro-oxidant state, TAC reflects the total antioxidant state, and OSI is a marker that reflects the overall OS [49]. Like in other studies analyzing OS in inflammation in the INFL animals, TOS and OSI were increased and TAC was reduced [50]. Our study showed that diclofenac and trolox significantly reduced TOS and OSI, without influencing TAC. *A. dracunculus* had a dose-dependent inhibitory effect on TOS and OSI, AD 25% having the lowest activity, and all AD dilutions reduced TOS and OSI less than trolox. All *A. abrotanum* dilutions had a lower inhibitory activity upon TOS and OSI and the effect was smaller than those of diclofenac, a nonsteroidal anti-inflammatory drug, and trolox, a synthetic antioxidant.

Vital cellular molecules, like DNA, proteins, and membrane lipids, are targets for oxidative attack [2]. DNA damage leads to mutations, genetic instability, and epigenetic changes. We investigate the association of OS with oxidative DNA damage by measuring 8-OHdG, an adduct of oxidative DNA damage, and the most reliable biomarker for this process [24,33]. In this study, in INFL animals, the levels of 8-OHdG were significantly increased compared to control animals. Many studies indicated that there is an increased level of 8-OHdG in many degenerative diseases and cancer types too [51,52]. *A. dracunculus* and AA extracts reduced 8-OHdG significantly, indicating that these extracts are good DNA oxidative injury inhibitors. For *A. dracunculus*, these effects were smaller than those of diclofenac but for *A. abrotanum*, the inhibitory activity on 8-OHdG was better than that of diclofenac. Like in other studies, oxidative DNA damage reduction was correlated with TOS reduction [53] for both extracts, namely *A. dracunculus* and *A. abrotanum*.

Proteins are structural and functional molecules that control cellular activity. Oxidative damage to proteins may result in protein dysfunction and structural alterations. AOPPs are heterogeneous compounds formed by the reaction of chlorinated oxidants with plasma proteins and are used as markers of plasma protein oxidative damage [54]. Furthermore, previous studies found that AOPPs activated the NF-κB-dependent pathway [55] and significantly increased ROS production by sensitizing NADPH oxidase. In this way, AOPPs increase inflammatory responses and AOPP reduction is a possible new therapeutic strategy to reduce the risk of complications in inflammatory diseases [56]. The present study identified increased levels of AOPPs in rats with inflammation. *A. dracunculus* reduced AOPPs in a dose-dependent way, with higher concentrations being more efficient. *A. abrotanum* had a smaller inhibitory effect on AOPPs and it was not dose-dependent. Both *A. dracunculus* and *A. abrotanum* had a lower inhibitory activity on AOPPs than diclofenac and trolox.

The most common ROS that can affect lipids are hydroxyl and hydroperoxyl radicals. Oxidative damage to lipids leads to lipoperoxide (LPO) formation. LPO is mainly localized in the cell membrane and causes alteration of membrane properties [3]. Furthermore, a high rate of LPO formation induces apoptosis or necrosis. From the secondary products of LPO, MDA has been most extensively evaluated [7] because inflammation results in MDA accumulation [57]. MDA serves not only as a marker of lipids’ oxidative damage in vivo but also as an inducer of inflammatory responses, creating a vicious cycle like AOPP [3,57]. In agreement with those of other studies that evaluated MDA in inflammatory responses, our study showed that MDA was much higher in INFL than in CONTROL animals [58]. *A. dracunculus* reduced MDA in a dose-dependent way, with higher concentrations having better inhibitory activity. The effect of *A. dracunculus* effect was smaller than that of diclofenac and trolox. *A. abrotanum* extract had a lower inhibitory effect on MDA as compared to *A. dracunculus*. Considering that quercetin decreases MDA and that *A. dracunculus* and *A. abrotanum* extracts contain quercetin, this can be a mechanism of inhibitory activity [59].

The endogenous synthesis of NO is catalyzed by the nitric oxide synthase (NOS) isozymes. In mammals, there are three NOS isoforms: neuronal NOS (nNOS) and endothelial NOS (eNOS), which are constitutively expressed and produce a small amount of NO, and inducible NOS (iNOS) that is upregulated by cytokines and other inflammatory mediators and produces large amounts of NO. Lately, one more constitutively expressed isozyme has been identified: the mitochondrial NOS (mtNOS) [60]. In inflammation, NO produced by activated macrophages is an important mediator in the cytotoxic/cytostatic mechanism of nonspecific immunity. NO concentrations were measured indirectly via nitrite concentrations with a Griess assay [61]. In the INFL animals, NO was increased. This happened due to the excessive production of NO, which could induce cell damage and inflammation progression [3]. Both tested extracts, namely *A. dracunculus* and *A. abrotanum*, and diclofenac and trolox lowered NO synthesis. The effects of *A. dracunculus* and *A. abrotanum* on NO were not different from those of diclofenac and trolox. We presume that polyphenol inhibition of iNOS expression [3] caused the reduction in NO production, and this effect can be useful in treating inflammatory diseases.

NO is a highly reactive gaseous free radical, which can be further oxidized to form RNS. Abnormal NO metabolism is involved in the pathogenesis of inflammation, sepsis, cancers, and many other diseases [60]. RNS such as peroxynitrite (ONOO^-^) mediate protein tyrosine nitration, resulting in 3-nitrotyrosine (3NT), a marker of OS [57]. INFL animals had a high 3NT concentration, and *A. dracunculus* and AA treatment lowered 3NT. Diclofenac and trolox reduced 3NT by decreasing both NO and ROS synthesis. *A. dracunculus* and *A. abrotanum* extract’s effect on 3NT was similar to that of trolox and better than that of diclofenac. Our results suggest that the inhibitory effects of *A. dracunculus* and *A. abrotanum* on NO production could contribute to OS decrease.

Because TAC provides a means of antioxidant activity, lacking detailed information about the composition and concentrations of specific antioxidants, specific endogenous nonenzymatic antioxidants can be used as biomarkers. It is well documented that the main contributor to plasmatic TAC is uric acid and thiols (SH) are the second biggest component contributing to TAC [61]. SH are sulfhydryl-containing organic compounds and important components of the plasma antioxidant system [62]. Inflammation reduced SH and only A. *abrotanum* treatment increased SH levels, but without influencing TAC. *A. dracunculus*, diclofenac, and trolox had no significant effect on SH.

Inflammatory cells release mediators that can stimulate transcription factors like NF-κB, which is involved in innate and adaptive immune functions, stress responses, cell proliferation, and cell death. NF-κB is a heterodimer composed of p50 and p65 subunits. In unstimulated cells, complexes with IκB are sequestered in the cytoplasm. Upon stimulation by an extracellular stimulus, NF-κB p65 is released and translocated into the nucleus, which activates the transcription of target genes, like iNOS and proinflammatory cytokines [63]. The beneficial effects of many natural compounds have been attributed to NF-κB p65 signaling reduction [64]. In our INFL animals, NF-κB p65 was significantly increased. A study analyzing *A. annuae* herba extracts has proven that it has anti-inflammatory activity by blocking the activation of the NF-κB pathway [2]. By measuring NF-κB p65 after *A. dracunculus* and *A. abrotanum* treatments, we obtained a similar result because both *A. dracunculus* and *A. abrotanum* reduced NF-κB p65 but the effect was smaller than those of diclofenac or trolox. This is an important anti-inflammatory mechanism of the tested extracts.

In inflammation, PAMPs or DAMPs stimulate NF-kB-dependent NLRP3 inflammasome activation [46,65]. The NLRP3 inflammasome is a multiprotein complex consisting of a leucine-rich repeat nucleotide-binding domain (NLR), pyrin domain containing receptor 3, plus apoptosis-associated speck-like protein containing a caspase recruitment domain (ASC), and procaspase-1. The result of NLRP3 inflammasome activation is caspase-1 activation and the secretion of IL-1β and IL-18 [66]. Furthermore, by releasing these pro-inflammatory mediators together with GSDMD, pyroptosis will be triggered [10,54]. Because sustained activation of NLRP3 inflammasome results in progression to chronic inflammatory diseases, autoimmune diseases, cardiometabolic diseases, cancer, neurological disorders, and others, NLRP3 inflammasome inhibition in acute phases became a therapeutic target [9,65,67,68]. The determination of serum IL-1β, IL-18, and GSDMD is commonly used when inhibitors of inflammasome activation are tested [67]. In our study, INFL animals had a high level of IL-1β, IL-18, and GSDMD and diclofenac caused a significant reduction. Non-steroidal anti-inflammatory drugs have been previously shown to inhibit the NLRP3 inflammasome in rodent models [69]. The trolox effect is probably through the antioxidant activity that reduced OS-induced inflammation. Treatment with *A. dracunculus* had a dose-dependent effect on IL-1β and IL-18, with AD 100% and AD 50% having a stronger inhibitory activity. *A. abrotanum* reduced IL-1β and IL-18 too, and the effect was not dose-dependent. The effects of both extracts were comparable to that of trolox and it may be due to the antioxidant activity of *A. dracunculus* and *A. abrotanum*. On GSDMD, *A. dracunculus* and *A. abrotanum* had a small inhibitory activity and this was smaller than those of diclofenac and trolox.

To evaluate the hepatic and renal toxicity, we added AST and ALT tests for liver injury and creatinine and urea for renal dysfunction [70]. The liver is a target of uncontrolled inflammation [71] because proinflammatory cytokines impair hepatocellular function. AST with normal rat values of 34–109 U/L and ALT with normal rat values of 13–56 U/L [70] were not influenced in the INFL animals, indicating that it was not an excessive inflammatory response. *A. dracunculus* and *A. abrotanum* extracts had no hepatotoxicity because both did not increase the transaminases.

It is a fact that the kidneys are particularly vulnerable to inflammation and a dysregulated inflammatory response can lead to renal dysfunction. First, the kidneys receive the entire blood volume without having anti-inflammatory defense mechanisms. Secondly, there are many inflammatory mediators in the renal tubules. Third, OS can also cause direct renal cell damage [72]. INLF animals and those treated with *A. dracunculus* and *A. abrotanum* had no significant changes in blood creatinine (normal rat value and urea).

## 5. Conclusions

The data of this study showed that *A. dracunculus* and *A. abrotanum* have in vitro and in vivo antioxidant effects by reducing free radical generation and therefore can be a therapeutic agent against OS-triggered diseases. The antioxidant activity is related to the significant content of polyphenols and flavonoids. Furthermore, the results also proved that *A. dracunculus* and *A. abrotanum* extracts can reduce the inflammatory response by decreasing NLRP3 inflammasome activation and OS-induced inflammation. Considering that, after a prolonged time, inflammation associated with OS creates a pathological vicious circle, which may lead to chronic diseases and cancer, we consider that *A. dracunculus* and *A. abrotanum* might be useful adjuvant antioxidant and anti-inflammatory therapies. Moreover, even when *A. dracunculus* and *A. abrotanum* had a smaller antioxidant and anti-inflammatory activity than diclofenac and trolox, due to their low toxicity and low costs, these plant extracts can be preferred to synthetic drugs, although further studies in this field are needed.

## Figures and Tables

**Table 1:** Total polyphenol content and total flavonoid content of *A. dracunculus* and *A. abrotanum* ethanolic extracts.

Plant Extract	Total Polyphenols Content (mg GAE/g d.w. Plant Material)	Total Flavonoids Content (mg QE/100g d.w. Plant Material)
*A. dracunculus*	2.26 ± 0.21	181.50 ± 32.10
*A. abrotanum*	2.10 ± 0.09	233.15 ± 18.64

**Table 2:** Liquid Chromatography–Diode Array Detection–Electro-Spray Ionization Mass Spectrometry phenolic compound tentative identification from *A. dracunculus* and *A. abrotanum* ethanolic extracts.

Peak No	R_t_ (min)	UV λ_max_ (nm)	[M + H]^+^ (*m*/*z*)	Compound Tentative Identification	Concentration (μg/g)	
					*A. dracunculus*	*A. abrotanum*
	3.22	275	154.1	3.5-dihydroxybenzoic acid	40.04 ± 2.02	57.07 ± 2.97
	11.04	323	355.1	3-caffeoylquinic acid (neochlorogenic acid)	233.10 ± 19.99	295.38 ± 14.60
	12.57	323	355.1	5-caffeoylquinic acid (chlorogenic acid)	3260.20 ± 99.41	3834.38 ± 56.00
	12.95	323	355.1	4-caffeoylquinic acid (criptochlorogenic acid)	536.16 ± 19.96	208.88 ± 11.70
	13.21	323	343.1	caffeoyl acid-glucoside	-	1022.06 ± 47.01
	13.72	324	194.1	iso-ferulic acid	409.99 ± 18.92	89.80 ± 3.89
	14.24	325	312.3	caffeoyl tartaric acid	474.28 ± 18.14	108.70 ± 7.14
	14.64	330/270	565.2	apigenin-arabinosyl-glucoside	30.22 ± 1.04	46.26 ± 1.94
	15.02	324	369.1	3-feruloylquinic acid	205.65 ± 16.33	196.41 ± 10.01
	15.47	324	369.1	4-feruloylquinic acid	686.28 ± 19.60	234.74 ± 9.97
	15.88	324	369.1	5-feruloylquinic acid	1442.56 ± 39.97	-
	16.03	255/360	611.3	quercetin-rutinoside (rutin)	606.67 ± 29.75	903.68 ± 45.51
	16.66	330/270	565.2	apigenin-glucosyl-arabinoside	197.63 ± 6.26	193.67 ± 10.14
	17.13	240/350	625.3	isorhamnetin-rutinoside	354.68 ± 28.21	240.37 ± 21.13
	17.52	323	517.1	3.4-dicaffeoylquinic acid	722.48 ± 36.70	1320.08 ± 60.21
	17.86	323	517.1	3.5-dicaffeoylquinic acid	1642.81 ± 65.57	2303.19 ± 85.22
	18.11	323	517.1	quinic acid derivative	-	443.79 ± 20.01
	18.35	323	517.1	4.5-dicaffeoylquinic acid	1403.42 ± 75.49	1458.02 ± 86.65
	18.73	324	531.1	3-feruloyl-4-caffeoylquinic acid	134.85 ± 4.36	592.24 ± 26.45
	19.42	324	531.1	4-feruloyl-5-caffeoylquinic acid	172.44 ± 4.99	325.77 ± 13.54
	19.84	324	545.1	3.4-diferuloylquinic acid	546.52 ± 20.17	164.61 ± 5.32
	20.44	324	545.1	3.5-diferuloylquinic acid	104.69 ± 4.03	191.50 ± 6.01
	21.32	324	531.1	5-caffeoyl-4-feruloyl-quinic acid	81.66 ± 6.36	237.03 ± 10.59
	22.91	323	679.1	3.4.5-tricaffeoylquinic acid	1188.49 ± 79.35	126.08 ± 9.01
	23.68	330/270	361.3	3.5-dihydroxy-6.7.4′-trimethoxyflavone	57.39 ± 6.04	45.16 ± 4.01
	24.39	330/270	375.3	3.5-dihydroxy-6.7.3′.4′-tetramethoxyflavone	35.40 ± 2.92	36.92 ± 2.85
				Total Phenolics	14,567.61 ± 622.65	14,675.80 ± 571.85

**Table 3:** In vitro antioxidant activity of the *A. dracunculus* and *A. abrotanum* ethanolic extracts.

Samples	DPPH μg TE/mL	FRAP μg TE/mL	H_2_O_2_ μg TE/mL	NO μg QE/mL
*A. dracunculus*	69.87 ± 1.41	67.04 ± 0.59	63.94 ± 0.47	63.41 ± 0.35
*A. abrotanum*	69.27 ± 0.25	67.13 ± 0.15	59.43 ±0.27	56.66 ± 0.34

DPPH—DPPH free radical scavenging capacity; FRAP—ferric reducing antioxidant power; H_2_O_2_—hydrogen peroxide scavenging capacity; NO—nitric oxide radical scavenging assay; TE—TROLOX equivalent; QE—quercitin equivalent.

**Table 4:** In vivo antioxidant activity of the *A. dracunculus* and *A. abrotanum* ethanolic extracts.

Groups	TOS (µmol H_2_O_2_ Equiv./L)	TAC (mmol Trolox Equiv./L)	OSI	8-OHdG (ng/mL)	AOPP (µmol/L)	MDA (nmol/L)	NO (µmol/L)	3-NT (ng/mL)	SH (µmol/L)
CONTROL	18.05 ± 3.74	1.09 ± 0.00	18.05 ± 3.74	69.46 ± 43.13	34.86 ± 6.47 ^#^	2.284 ± 0.05	22.35 ± 6.52	20.98 ± 2.75	324.67 ± 22.21 ^##^
INFL	45.56 ± 13.57 ***	1.09 ± 0.00	41.93 ± 12.49 ***	77.39 ± 33.53	49.15 ± 11.09 **	3.349 ± 0.90 *	39.93 ± 2.59 *	63.22 ± 9.11 ***	263 ± 46.08 **
AD 100%	18.80 ± 4.65 ^#^	1.09 ± 0.00	17.29 ± 4.29 ^#^	32.76 ± 22.71	34.54 ± 5.89 ^##^	2.126 ± 0.19 ^##^	27.61 ± 5.16 ^#^	25.89 ± 1.21 ^###^	263 ± 31.80 ^#^
AD 50%	22.13 ± 7.72 ^###^	1.09 ± 0.00	20.37 ± 7.11 ^###^	41.91 ± 18.33	31.76 ± 8.34 ^##^	2.141 ± 0.33 ^##^	21.48 ± 5.00 ^#^	29.66 ± 5.30 ^###^	275.22 ± 96.02
AD 25%	34.43 ± 11.16	1.09 ± 0.00	31.70 ± 10.29	31.58 ± 5.23 ^##^	32.60 ± 12.81 ^##^	2.357 ± 0.36 ^##^	35.52 ± 8.37	31.67 ± 8.43 ^##^	269.22 ± 85.36
AA 100%	25.70 ± 10.11 ^##^	1.09 ± 0.00	23.66 ± 9.31 ^##^	31.78 ± 14.08 ^###^	35.14 ± 14.38 ^#^	2.341 ± 0.31 ^#^	30.41 ± 6.75 ^#^	25.97 ± 9.93 ^###^	321.57 ± 44.42 ^##^
AA 50%	27.53 ± 6.31 ^###^	1.09 ± 0.00	25.35 ± 5.81 ^##^	32.24 ± 35.11 ^###^	33.74 ± 12.36 ^#^	2.578 ± 0.37 ^#^	30.81 ± 3.40 ^#^	29.77 ± 9.71 ^###^	226.33 ± 34.87
AA 25%	26.73 ± 10.39 ^###^	1.09 ± 0.00	24.63 ± 9.57 ^##^	32.48 ± 7.64 ^###^	41.82 ± 7.45 ^#^	2.692 ± 0.32 ^#^	31.03 ± 5.12 ^#^	30.06 ± 8.10 ^###^	203.89 ± 14.53
TX	15.33 ± 5.09 ^#^	1.09 ± 0.00	14.10 ± 4.68 ^#^	33.36 ± 8.64 ^###^	29.81 ± 7.51 ^###^	2.817 ± 0.30	40.68 ± 7.05	27.63 ± 8.53	278.56 ± 34.06
DICLO	19.47 ± 2.07 ^##^	1.09 ± 0.00	17.94 ± 1.91	60.61 ± 20.18 ^###^	24.77 ± 5.70 ^##^	3.215 ± 0.10	27.52 ± 5.38	35.56 ± 3.17	243 ± 37.36

Vs CONTROL: * *p* < 0.05; ** *p* < 0.01; *** *p* < 0.001; Vs INFL: ^#^ *p* < 0.05; ^##^ *p* < 0.01; ^###^ *p* < 0.001; INFL—inflammation group; AD 100%—*A. dracunculus* 100%; AD 50%—*A. dracunculus* 50%; AD 25%—*A. dracunculus* 25%; AA 100%—*A. abrotanum* 100%; AA 50%—*A. abrotanum* 50%; AA 25%—*A. abrotanum* 25%; TX—Trolox; DICLO—diclofenac; TOS—Total oxidative status; TAC—Total antioxidant capacity; OSI—Oxidative stress index; 8-OHdG—8-hydroxydeoxyguanosine; AOPP—Advanced oxidation protein products; MDA: Malonyldialdehide; NOx—Nitrites and nitrates; 3NT—3-nitrotyrosine; SH—total thiols.

**Table 5:** Inflammation biomarkers of the study groups.

Groups	NfkB-p65 (ng/mL)	IL-1b (pg/mL)	IL-18 (pg/mL)	Gasdemine D (ng/mL)
CONTROL	141.39 ± 5.61	24.58 ± 2.59	16.31 ± 9.86	5.69 ± 0.43
INFL	247.58 ± 23.76 ***	56.04 ± 6.82 ***	59.58 ± 9.22 ***	9.50 ± 1.65 **
AD 100%	154.38 ± 16.64 ^###^	26.67 ± 3.26 ^###^	23.16 ± 9.32 ^###^	7.60 ± 1.52 ^#^
AD 50%	159.42 ± 19.15 ^###^	28.33 ± 3.54 ^###^	25.20 ± 4.71 ^###^	7.38 ± 0.49 ^#^
AD 25%	152.88 ± 24.84 ^###^	44.58 ± 4.22 ^#^	41.95 ± 5.20 ^#^	7.83 ± 1.17 ^#^
AA 100%	154.75 ± 19.90 ^###^	20.63 ± 3.75 ^###^	23.93 ± 4.85 ^###^	6.96 ± 1.62 ^#^
AA 50%	172.86 ± 14.40 ^###^	30.00 ± 5.49 ^###^	26.52 ± 7.69 ^###^	6.66 ± 1.38 ^#^
AA 25%	163.47 ± 14.12 ^###^	30.00 ± 3.19 ^###^	24.94 ± 9.32 ^###^	6.81 ± 2.07 ^#^
TX	153.06 ± 9.52 ^###^	29.37 ± 6.25 ^###^	26.32 ± 8.57 ^###^	6.46 ± 0.53 ^##^
DICLO	138.52 ± 9.47 ^###^	27.50 ± 6.35 ^###^	21.37 ± 5.32 ^###^	5.32 ± 1.87 ^##^

Vs CONTROL: ** *p* < 0.01; *** *p* < 0.001; Vs INFL: ^#^ *p* < 0.05; ^##^ *p* < 0.01; ^###^ *p* < 0.001; INFL—inflammation group; AD 100%—*A. dracunculus* 100%; AD 50%—*A. dracunculus* 50%; AD 25%—*A. dracunculus* 25%; AA 100%—*A. abrotanum* 100%; AA 50%—*A. abrotanum* 50%; AA 25%—*A. abrotanum* 25%; TX—Trolox; DICLO—diclofenac; NfkB-p65—Nuclear factor-κB; IL-1b—Interleukine 1-b; IL-18—Interleukine 18.

**Table 6:** Liver and renal injury biomarkers of the study groups.

Groups	ALT	AST	Creatinine mg/dL	Ureea mg/dL
CONTROL	43.33 ± 18.5	49.01 ± 13.5	0.74 ± 0.04	33.50 ± 2.44
INFL	46.82 ± 11.2	48.62 ± 13.1	0.86 ± 0.25	40.43 ± 9.02
AD 100%	42.03 ± 6	42.56 ± 8.2	0.82 ± 0.09	39.48 ± 6.9
AD 50%	39.21 ± 8.5	41.13 ± 7	0.79 ± 0.15	43.05 ± 6.97
AD 25%	40.06 ± 13.0	39.04 ± 6.5	0.94 ± 0.19	43.65 ± 18.56
AA 100%	43.78 ± 14.6	43.26 ± 9	0.82 ± 0.20	40.73 ± 8.07
AA 50%	41.22 ± 12.5	41.20 ± 9.8	0.71 ± 0.16	38.89 ± 5.96
AA 25%	29.14 ± 7.6 ^#^	26.62 ± 5.9 ^#^	0.80 ± 0.28	40.86 ± 13.37
TX	37.23 ± 7.6	35.8 ± 6.6	0.79 ± 0.17	37.48 ± 5.20
DICLO	32.12 ± 5.8	32.86 ± 10.7	0.74 ± 0.22	40.42 ± 2.35

Vs INFL: ^#^ *p* < 0.05; INFL—inflammation group; AD 100%—*A. dracunculus* 100%; AD 50%—*A. dracunculus* 50%; AD 25%—*A. dracunculus* 25%; AA 100%—*A. abrotanum* 100%; AA 50%—*A. abrotanum* 50%; AA 25%—*A. abrotanum* 25%; TX—Trolox; DICLO—diclofenac; AST—aspartat aminotransferase; ALT—alanin aminotransferase.

## Data Availability

Dataset available on request from the authors.
